# BRD4 Inhibition alleviates sepsis-associated acute kidney injury via suppression of NOX4-mediated oxidative stress and inflammation

**DOI:** 10.1038/s41420-026-03113-y

**Published:** 2026-04-21

**Authors:** Jing Jia, Kangkang Ji, Yong Zhou, Yang Huang, Ang Li, Fan Ye, Wen Huang, Fang Chen, Guoyuan Lu

**Affiliations:** 1 Department of Nephrology, The First Affiliated Hospital of Soochow University, Suzhou, China; 2 Department of Nephrology, Yancheng First Hospital, Affiliated Hospital of Nanjing University Medical School, The Yancheng Clinical College of Xuzhou Medical University, Yancheng Clinical Medical College of Jiangsu University, The First People’s Hospital of Yancheng, Yancheng, China; 3 Department of Respiratory and Critical Care Medicine, Binhai County People’s Hospital, Yancheng, China; 4 College of Biomedicine and Health, Huazhong Agricultural University, Wuhan, China; 5 Department of General Surgery, Yancheng First Hospital, Affiliated Hospital of Nanjing University Medical School, The Yancheng Clinical College of Xuzhou Medical University, Yancheng Clinical Medical College of Jiangsu University, The First People’s Hospital of Yancheng, Yancheng, China; 6 Department of Gastroenterology, The Yancheng Clinical College of Xuzhou Medical University, Yancheng, China; 7 Department of Critical Care Medicine, The Yancheng Clinical College of Xuzhou Medical University, Yancheng, China; 8 Yancheng Key Laboratory of Molecular Epigenetics, Yancheng Medical Research Center of Nanjing University Medical School, The First People’s Hospital of Yancheng, Yancheng, China; 9 Department of Urology, The Yancheng Clinical College of Xuzhou Medical University, The First people’s Hospital of Yancheng, Yancheng, China

**Keywords:** Acute kidney injury, Transcriptional regulatory elements

## Abstract

Sepsis-associated acute kidney injury (S-AKI) is characterized by complex pathological mechanisms, primarily driven by oxidative stress and inflammation, with NADPH oxidase 4 (NOX4) playing a critical role. However, the regulatory mechanisms underlying NOX4 activation remain incompletely understood. In this study, we found that circulating levels of NOX4 and the chromatin acetylation “reader” bromodomain-containing protein 4 (BRD4) were significantly elevated in S-AKI patients and positively correlated with renal dysfunction markers. These clinical findings were further validated in both LPS-induced and cecal ligation and puncture (CLP)-induced S-AKI models, in which BRD4 and NOX4 were markedly upregulated in the kidney. Prominent induction was observed in renal tubular epithelial cells, and this upregulation was associated with exacerbated inflammation, oxidative stress, and renal injury. Pharmacological inhibition of NOX4 effectively mitigated these pathological changes in both models. Similarly, treatment with the BRD4 inhibitor JQ1 conferred renoprotection and downregulated NOX4. Mechanistically, chromatin immunoprecipitation assays demonstrated that upon LPS stimulation, BRD4 is recruited to the NOX4 promoter, facilitates the co-recruitment of the histone acetyltransferase P300, and promotes local histone H3 acetylation to directly activate NOX4 transcription. Importantly, NOX4 overexpression delivered by AAV, which was predominantly detected in renal tubules, largely abolished the protective effects of JQ1, indicating that NOX4 is a critical downstream target of BRD4. In conclusion, our findings identify the BRD4/P300/NOX4 transcriptional regulatory axis as a key pathogenic mechanism in S-AKI, offering a novel therapeutic insight for this condition.

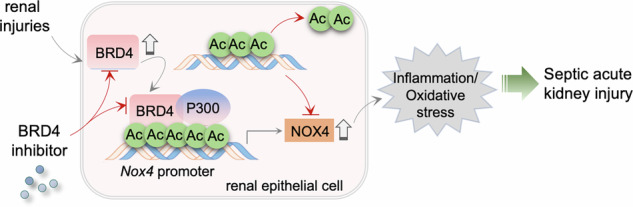

## Introduction

Sepsis-associated acute kidney injury (S-AKI) is a common and life-threatening clinical syndrome marked by a sudden decline in glomerular filtration rate, elevated serum creatinine, or oliguria in the setting of sepsis, with hallmark features including excessive inflammatory response, oxidative stress, microcirculatory dysfunction, and mitochondrial impairment [1, 2]. Unfortunately, S-AKI is strongly associated with poor clinical outcomes [3], and current therapies can only slow but not halt or reverse the progression of septic acute kidney injury. However, the precise molecular mechanisms underlying S-AKI remain poorly defined, resulting in a lack of effective and targeted therapeutic strategies [4].

Oxidative stress and inflammation are central to S-AKI pathophysiology. During sepsis, the host response triggers a surge of inflammatory mediators and reactive oxygen species (ROS), leading to widespread endothelial injury and microvascular dysfunction [5]. Nicotinamide adenine dinucleotide phosphate (NADPH) oxidase 4 (NOX4) is a constitutively expressed renal enzyme that is a major source of ROS [6], playing a pivotal role in modulating oxidative stress and downstream pro-inflammatory pathways [7, 8]. Accumulating evidence shows that NOX4 is critically involved in the development of AKI [9–12], and that pharmacological inhibition of NOX4 may represent a promising therapeutic strategy to attenuate oxidative damage and inflammation in S-AKI [9, 10]. Furthermore, recent studies suggest that NOX4 expression is regulated, at least in part, by epigenetic mechanisms involving histone acetylation [13–15], yet the upstream regulators of NOX4 in S-AKI remain to be elucidated.

The bromodomain and extra-terminal (BET) protein family functions as readers of histone acetylation, recognizing acetyl-lysine residues and thereby governing transcriptional programs [16, 17]. Histone acetyltransferases such as P300 serve as co-activators by depositing these acetyl marks [18], which in turn recruit BET proteins [19]. The BET family comprises four members - BRD2, BRD3, BRD4 and the testis-specific BRDT [20] - of which BRD4 is the most extensively characterized. BRD4 has been implicated in various oxidative stress and inflammatory diseases, including cardiac hypertrophy [21], chronic kidney disease [22], and acute kidney injury [23]. Notably, BRD4 has been shown to regulate NOX4 expression in contexts such as myofibroblast differentiation [24], cardiac hypertrophy [15], lung fibrosis [25], and renal fibrosis [26], but its role in epigenetically controlling NOX4 in S-AKI has not been defined.

In the present study, we explore the role and underlying mechanisms by which BRD4 promotes NOX4 transcription, oxidative stress, and inflammation during S-AKI. We hypothesize that targeting aberrant BRD4 activation may offer a novel therapeutic strategy for patients with S-AKI.

## Results

### Characteristics of the study population

The study enrolled 17 patients with sepsis-associated acute kidney injury (S-AKI) and 10 healthy volunteers. Key differences in demographics, organ function, and sepsis severity were observed between the groups (Table 1). S-AKI patients were significantly older than controls (54.06 ± 19.32 vs. 40.80 ± 17.29 years, *p* = 0.043), with no difference in sex distribution (41.2% vs. 70% male, *p* = 0.148). Renal function was severely impaired in S-AKI patients, evidenced by elevated serum creatinine (CREA) (191.30 vs. 68.45 μmol/L, *p* < 0.001) and UREA (25.79 ± 9.58 vs. 5.14 ± 1.07 mmol/L, *p* < 0.001). Laboratory findings confirmed a sepsis-associated profile, including thrombocytopenia (61.0 vs. 231.5 × 10^9^/L, *p* = 0.011), leukocytosis (14.20 vs. 6.08 × 10^9^/L, *p* < 0.001), elevated bilirubin (27.20 vs. 13.39 μmol/L, *p* < 0.001), neutrophilia (13.19 vs. 2.58 × 10^9^/L, *p* < 0.001), and lymphopenia (0.67 vs. 2.66 × 10^9^/L, *p* < 0.001). The patients exhibited high sepsis severity, with a median SOFA score of 10, impaired oxygenation (P/F ratio: 245), depressed consciousness (GCS: 11), and markedly elevated procalcitonin (71.49 ng/mL).

**Table 1:** Baseline clinical characteristics of the study population.

Characteristic	Healthy Controls (*n* = 10)	S-AKI Patients (*n* = 17)	χ2/t/Z	*p*-value
**Demographics**				
Age (years)	40.80 ± 17.29	54.06 ± 19.32	1.787	0.043
Sex (Male, n, %)	7 (70%)	7 (41.2%)	2.095	0.148
**Renal function**				
Creatinine (μmol/L)	68.45 (55.63, 69.25)	191.30 (130.10, 328.85)	−4.268	<0.001
UREA(mmol/L)	5.14 ± 1.07	25.79 ± 9.58	6.742	<0.001
**Routine laboratory tests**				
Platelet count (PLT, ×10⁹/L)	231.5 (204.0, 317.0)	61.0 (29.0, 112.0)	−2.536	0.011
Total Bilirubin (TBIL, μmol/L)	13.39 (12.27, 14.71)	27.20 (15.50, 57.20)	−3.414	<0.001
White Blood Cell count (WBC, ×10^9^/L)	6.08 (4.91, 6.55)	14.20 (12.23, 23.04)	−3.917	<0.001
Neutrophils (NEUT, ×10⁹/L)	2.58 (2.19, 3.35)	13.19 (10.66, 17.81)	−4.268	<0.001
Lymphocytes (ALC, ×10⁹/L)	2.66 (2.16, 3.21)	0.67 (0.35, 0.76)	−4.067	<0.001
**Sepsis severity scores**				
SOFA score	NA	10 (7, 12)	NA	NA
PaO₂/FiO₂ ratio (P/F)	NA	245 (215, 296)	NA	NA
Glasgow Coma Scale (GCS)	NA	11 (6, 14)	NA	NA
Procalcitonin (PCT, ng/mL)	NA	71.49 (24.71, 100.00)	NA	NA

This parameter was not measured in healthy volunteers as it is not part of routine health screening for asymptomatic individuals.

A PCT value > 100 ng/mL indicates severe systemic bacterial infection. For statistical analysis, values > 100 were treated as 100.

*NA* not applicable.

### Serum NOX4 and BRD4 levels are elevated and correlate with clinical indicators in patients with S-AKI

**Fig. 1:**
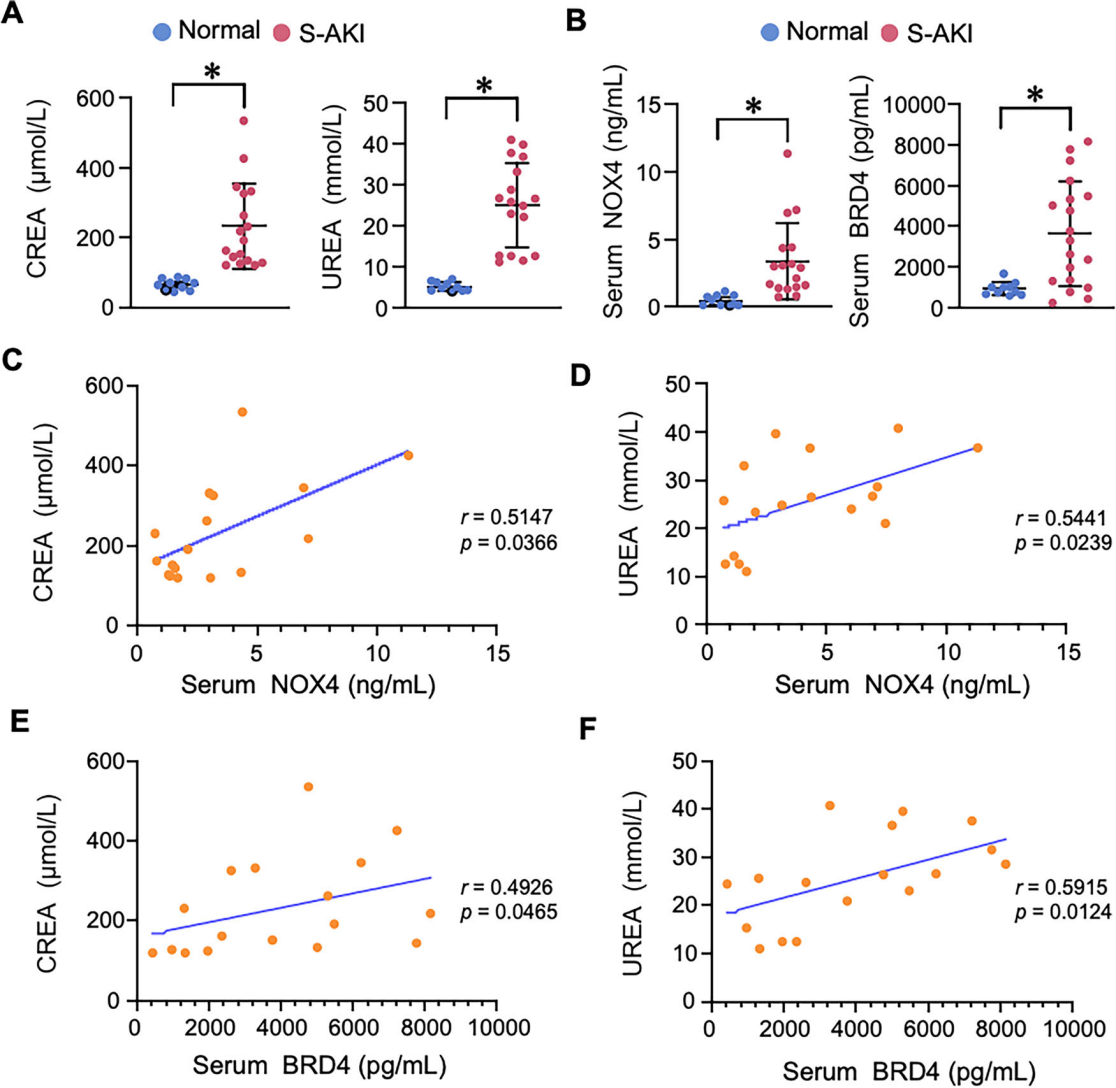
Serum NOX4 and BRD4 levels is upregulated and correlated with clinical indicators in patients with sepsis-associated acute kidney injury (S-AKI). **A** Examinations of CREA and UREA of normal persons (*n* = 10) and S-AKI patients (*n* = 17). Data were presented as means ± SEM, *P < 0.05, one-way ANOVA. **B** Examinations of serum NADPH oxidase 4 (NOX4) and bromodomain-containing protein 4 (BRD4) of normal persons (*n* = 10) and S-AKI patients (*n* = 17). Data were presented as means ± SEM, *P < 0.05, one-way ANOVA. **C** Correlation analysis of serum NOX4 expression with CREA in S-AKI patients. **D** Correlation analysis of serum NOX4 expression with UREA in S-AKI patients. **E** Correlation analysis of serum BRD4 expression with CREA in S-AKI patients. **F** Correlation analysis of serum BRD4 expression with UREA in S-AKI patients. Pearson’s test was used in **D** and **F** for correlation analysis. Spearman’s test was used in **C** and **E** for correlation analysis, *n* = 17, *P < 0.05.

Having characterized the clinical features of the enrolled participants, we next examined whether the expression of circulating NOX4 and BRD4 was altered in S-AKI. CREA and UREA levels were markedly elevated in S-AKI patients compared with healthy controls (Fig. 1A), confirming renal dysfunction in the patient cohort. ELISA analysis further revealed significantly increased levels of NOX4 and BRD4 in the serum of S-AKI patients compared to healthy controls (Fig. 1B). Correlation analysis showed that both NOX4 and BRD4 expressions were positively correlated with CREA and UREA levels (Fig. 1C–F). These findings suggest that the elevated levels of circulating NOX4 and BRD4 are associated with the impairment of renal function and may serve as potential biomarkers reflecting the severity of S-AKI.

### NOX4 and BRD4 are upregulated in the kidneys of S-AKI mice and associated with increased inflammation and oxidative stress

**Fig. 2:**
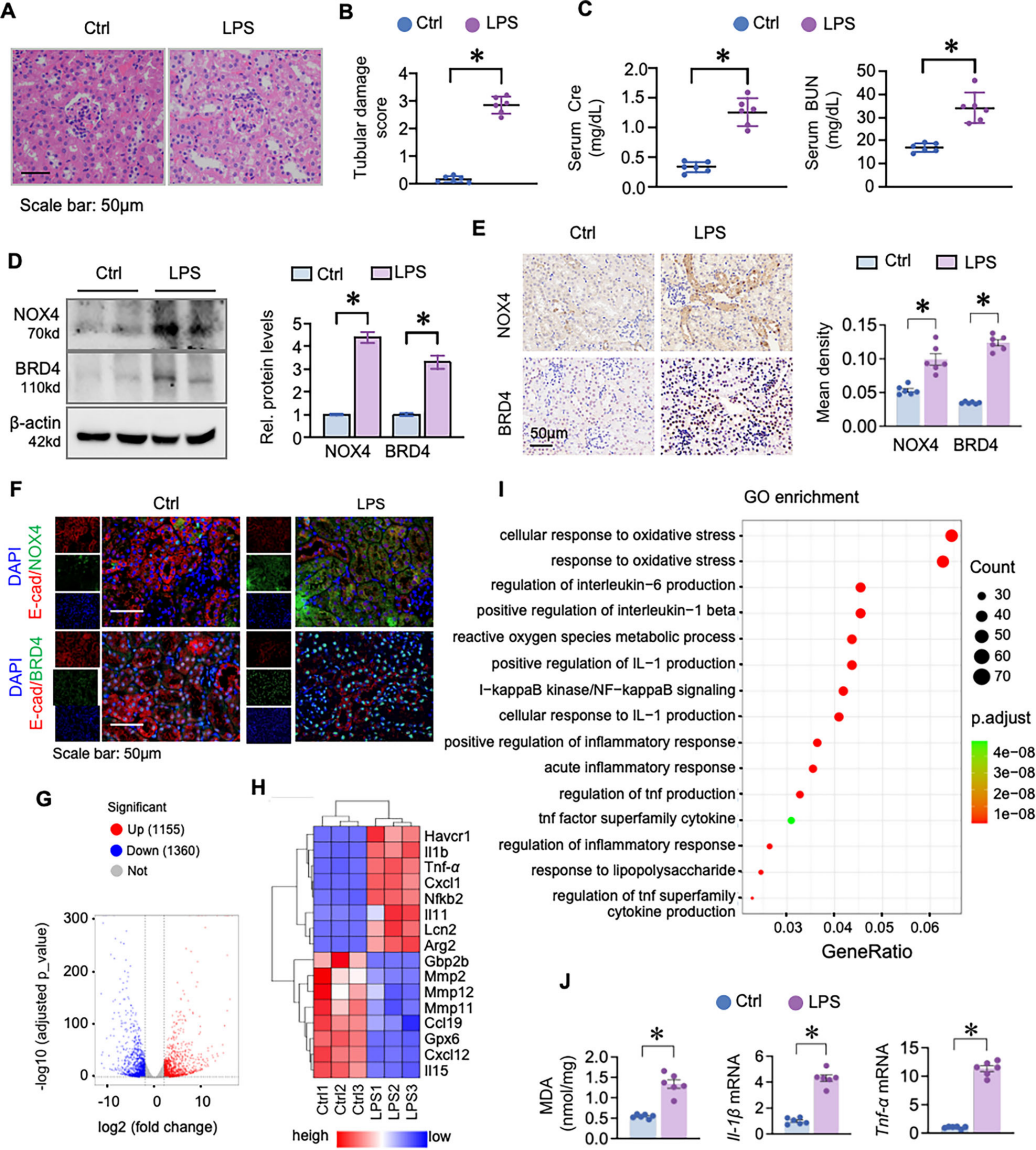
NOX4 and BRD4 expression as well as inflammation and oxidative stress were increased in LPS-induced AKI mouse model. C57BL/6 mice were subjected to LPS (10 mg/kg, intraperitoneally injection once) for 24 h (six mice in each group). **A** Representative photomicrographs of kidney sections stained with Hematoxylin-eosin staining (H&E) from Control (Ctrl) and LPS-treated mice. **B** Quantitation of tubular damage in (**A**). **C** Serum creatinine (Cre) and blood urine nitrogen (BUN) examinations. **D** Western blots of NOX4 and BRD4 from Ctrl and LPS-treated mice. Two randomly selected samples from each group were shown. The right panel is quantitation of Western blots in (**D**). **E** Representative photomicrographs of kidney sections from Ctrl and LPS-treated mice stained for NOX4 and BRD4 by immunohistochemistry (IHC) staining. The right panel is quantifications of positive staining areas of NOX4 and BRD4. **F** Representative IF staining for BRD4 or NOX4 (green) and the epithelial marker E-cadherin (E-cad, red) of kidney sections from Ctrl and LPS-treated mice. Nuclei were counterstained with DAPI (blue). **G** Volcano plot showing the differentially expressed genes in Ctrl and LPS-treated mice (*n* = 3). Significantly upregulated genes are shown in red, and significantly downregulated genes are shown in blue. All other genes are shown in gray. **H** Heatmap of significantly altered genes between Ctrl and LPS groups. **I** Gene ontology (GO) pathway enrichment analysis of the differentially expressed genes. **J** MDA examination, mRNAs of *IL-1β* and *TNF-α* in renal tissues from the experimental mice indicated above. Data were presented as means ± SEM. *P < 0.05, one-way ANOVA.

To investigate NOX4 and BRD4 expression in vivo, we utilized a well-established LPS-induced S-AKI mouse model, where systemic inflammation leads to kidney injury [27]. As anticipated, LPS-injected mice exhibited intracellular vacuolization and loss of brush border as demonstrated by Hematoxylin-eosin staining (H&E) (Fig. 2A, B). Serum creatinine (Cre) and blood urea nitrogen (BUN) levels were significantly elevated following LPS administration (Fig. 2C). Western blotting and immunohistochemistry further confirmed upregulation of NOX4 and BRD4 in kidneys following LPS treatment (Fig. 2D, E). To confirm the cellular localization of BRD4 and NOX4, we conducted immunofluorescence co-staining with the epithelial marker E-cadherin (E-cad). BRD4 and NOX4 signals were both largely colocalized with E-cad-positive tubular epithelial cells, suggesting that their upregulation during LPS-induced S-AKI is particularly prominent in tubular epithelial cells, although expression in other renal cell populations cannot be excluded (Fig. 2F). RNA-seq analysis of renal tissues identified 1,155 upregulated and 1,360 downregulated genes (Fig. 2G). Heatmap and GO analysis revealed marked enrichment of inflammation- and oxidative stress-related pathways (Fig. 2H, I). Consistently, levels of malondialdehyde (MDA, a marker of lipid peroxidation), as well as mRNA levels of proinflammatory cytokines (IL-1β and TNF-α) were significantly increased in LPS-treated kidneys (Fig. 2J). These findings indicate that NOX4 and BRD4 upregulation in S-AKI is closely associated with enhanced oxidative stress and inflammation.

### NOX4 and BRD4 upregulation is correlated with elevated inflammation and oxidative stress in LPS-treated HK2 cells

**Fig. 3:**
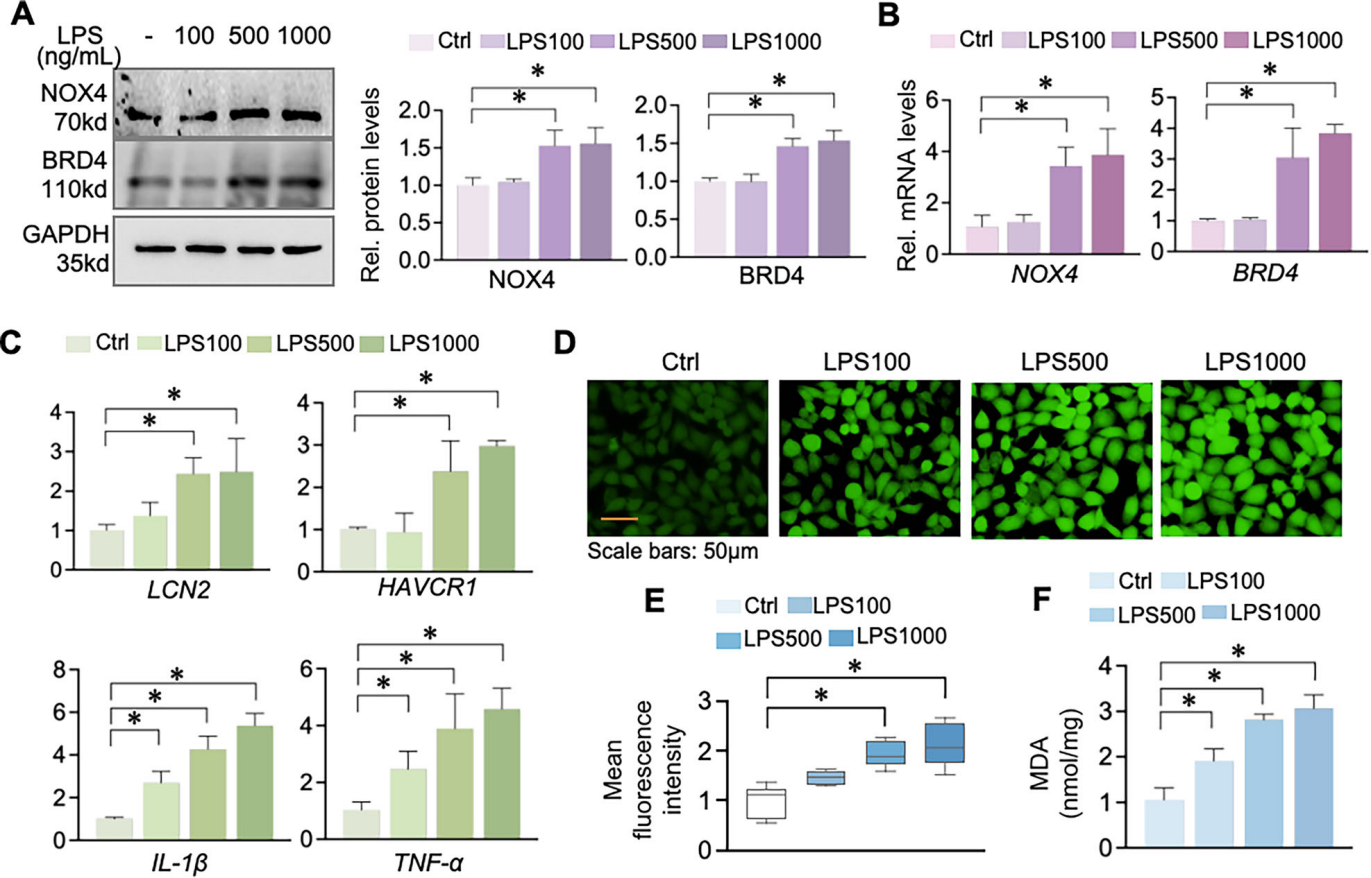
Upregulation of NOX4 and BRD4 is correlated with inflammation and oxidative stress increase in LPS-treated HK2 cells. **A** Western blots. HK2 cells were treated with increasing doses of LPS (100, 500, 1000 ng/mL) for 24 h and then assayed for NOX4 and BRD4. GAPDH served as internal control. Right panel is the quantifications. **B** Quantitative real-time PCR (qRT-PCR) analysis of *BRD4* and *NOX4* mRNAs from HK2 cells. **C** qRT-PCR detected the mRNA levels of of *LCN2*, *HAVCR1*, *IL-1β* and *TNF-α* in Control (Ctrl) and LPS-treated HK2 cells. **D** ROS detection using DCFH-DA (10 μM, 30 min) and representative ROS images captured with fluorescence microscopy. **E** Quantification of ROS levels in (**D**). **F** Malondialdehyde (MDA) examination of LPS-treated HK2 cells. Data were presented as means ± SD of three repeated cell assays. *P < 0.05; one-way ANOVA.

We next utilized LPS-challenged human renal tubular epithelial HK-2 cells as an in vitro S-AKI model. HK2 cells were treated with LPS at doses of 100 ng/mL, 500 ng/mL and 1000 ng/mL for 24 h. NOX4 and BRD4 protein and mRNA levels were significantly increased at 500 and 1000 ng/mL compared to control cells (Fig. 3A, B). Since there was no significant difference between the 500 and 1000 ng/mL groups, 500 ng/mL was chosen for subsequent experiments. LPS-treated HK-2 cells showed increased levels of renal injury markers neutrophil gelatinase-associated lipocalin (NGAL) and kidney injury molecule-1 (KIM-1) and proinflammatory cytokines IL-1β and TNF-α (Fig. 3C). The total ROS detected with the fluorescent dye DCFH-DA and MDA levels were also significantly increased in LPS-treated cells (Fig. 3D–F), indicating that NOX4 and BRD4 induction is associated with heightened oxidative stress and inflammation in tubular epithelial cells.

### Inhibition of NOX4 attenuates renal injury in S-AKI mice

**Fig. 4:**
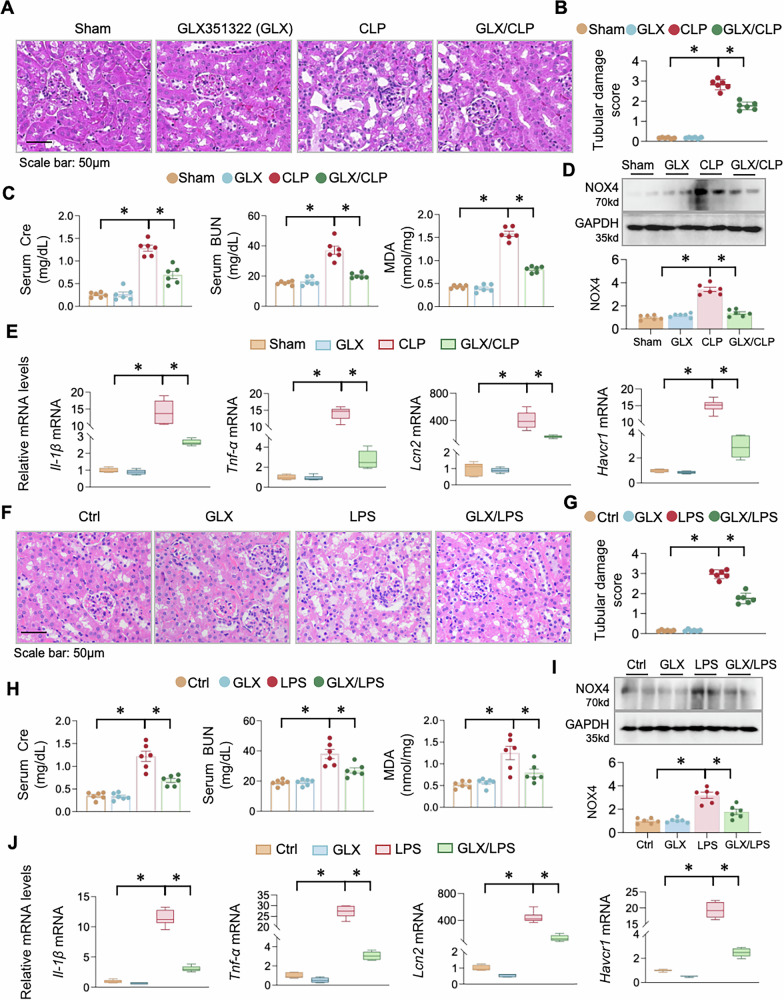
NOX4 inhibition protects against renal injury, inflammation, and oxidative stress in S-AKI mice. C57BL/6 mice were randomly divided into four groups (*n* = 6 per group): (1) Sham; (2) GLX351322 alone (GLX, 5 mg/kg, i.p. once); (3) CLP model; (4) GLX pretreatment of CLP mice. **A** Representative photomicrographs of kidney sections from above mice stained by H&E. **B** Quantification of tubular damage based on (**A**). **C** Cre, BUN and renal MDA levels were measured in the experimental mice indicated above. **D** Western blots. The renal tissues were assayed for NOX4. The lower panel was the quantitative analysis of the bands. **E** QPCR of *Il-1β, Tnf-α, Lcn2* and *Havcr1* mRNAs in renal tissues. C57BL/6 mice were randomly divided into four groups (*n* = 6 per group): (1) Vehicle control (Ctrl); (2) GLX351322 alone (GLX, 5 mg/kg, i.p. once); (3) LPS model; (4) GLX pretreatment of LPS mice. **F** Representative photomicrographs of kidney sections from above mice stained by Hematoxylin-eosin staining (H&E). **G** Quantification of tubular damage based on (**F**). **H** Serum creatinine (Cre), blood urine nitrogen (BUN) and renal malondialdehyde (MDA) levels were measured in the experimental mice indicated above. **I** Western blots. The renal tissues were assayed for NOX4. The lower panel was the quantitative analysis of the bands. **J** QPCR of *Il-1β, Tnf-α, Lcn2* and *Havcr1* mRNAs in renal tissues. Data were presented as means ± SEM, *P < 0.05, *n* = 6, two-way ANOVA.

To determine the functional role of NOX4 in S-AKI, we employed both LPS-induced and CLP-induced models and treated them with the NOX4-specific inhibitor GLX351322 (GLX), which reportedly inhibits hydrogen peroxide production with an IC50 value of 5 μM [28]. GLX alone had no adverse effect on kidney morphology but significantly alleviated tubular injury such as tubular dilation and brush border loss of CLP-induced mice (Fig. 4A, B) and LPS-induced mice (Fig. 4F, G). Consistent with the histopathological improvement, CLP-induced increases in serum Cre, BUN, and MDA were mitigated by GLX treatment (Fig. 4C). GLX also suppressed CLP-induced NOX4 protein upregulation (Fig. 4D) and reversed the mRNA induction of NGAL, KIM-1, IL-1β, and TNF-α (Fig. 4E). Similarly, in the LPS model, GLX treatment attenuated the elevation in Cre, BUN and MDA levels, suppressed NOX4 protein upregulation, and reduced the mRNA induction of key injury and inflammatory markers, including NGAL, KIM-1, IL-1β and TNF-α (Fig. 4H–J). Collectively, these results demonstrate that NOX4 inhibition confers robust protection against S-AKI by improving renal function, reducing oxidative stress, and suppressing inflammation.

### BRD4 inhibition attenuated NOX4 expression, inflammation, and oxidative stress in LPS-stimulated HK2 cells

**Fig. 5:**
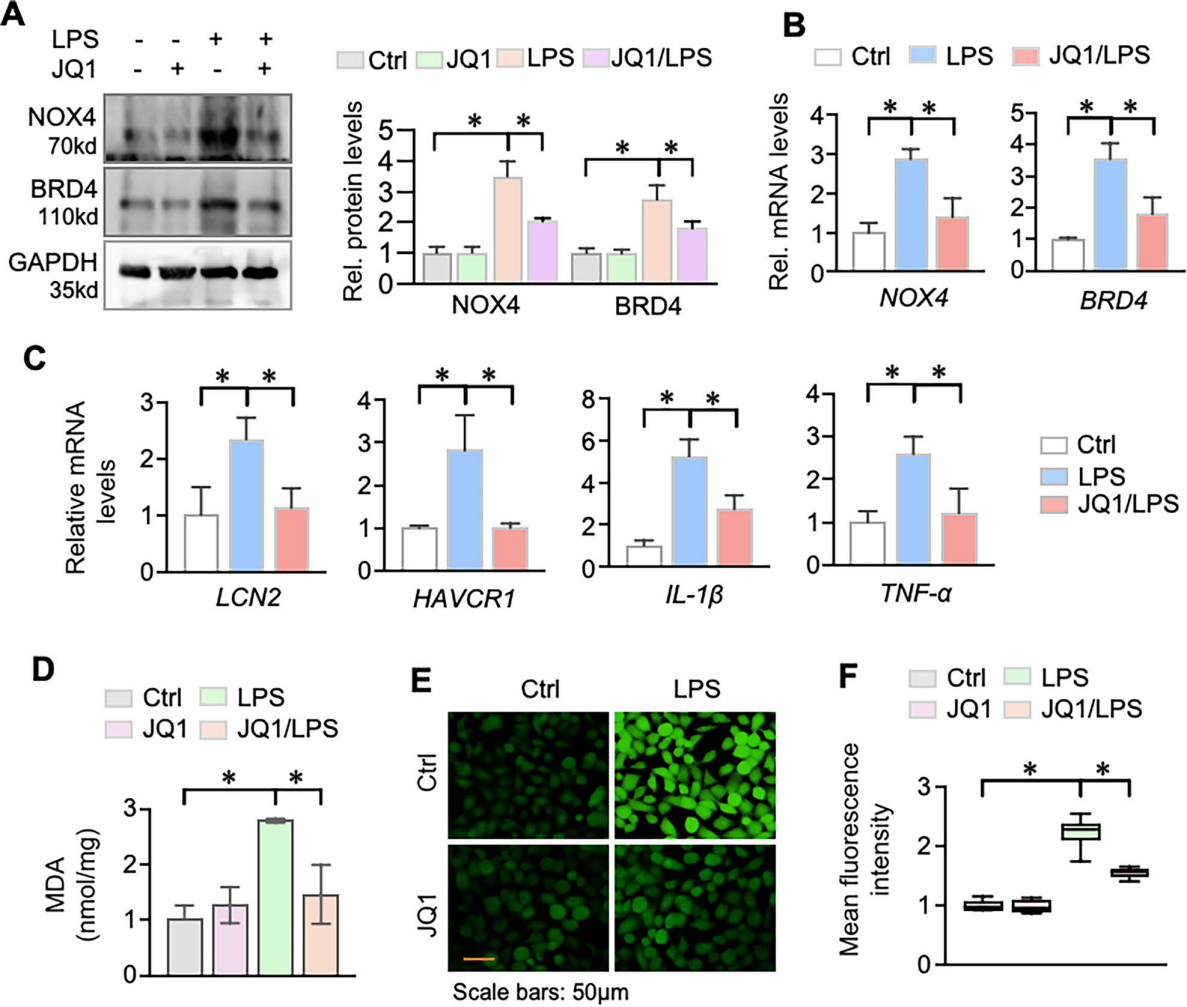
BRD4 inhibition attenuated NOX4 upregulation, inflammation and oxidative stress in LPS-stimulated HK-2 cells. **A** HK2 cells were treated with LPS (500 ng/mL) in presence or absence of JQ1 (10 μM) for 24 h, and then the cell lysates were tested for NOX4 and BRD4. The right panel is the quantification. **B** qRT-PCR of cells from Control (Ctrl), LPS and JQ1/LPS for *NOX4* and *BRD4* mRNAs. **C** qRT-PCR of cells from Control (Ctrl), LPS and JQ1/LPS for *LCN2*, *HAVCR1*, *IL-1β* and *TNF-α* mRNAs. **D** Malondialdehyde (MDA) examination in HK2 cells treated with LPS in presence or absence of JQ1 for 24 h. **E** ROS detection using DCFH-DA (10 μM, 30 min) and representative ROS images captured with fluorescence microscopy. **F** Quantification of ROS levels in (**E**). Data were presented as means ± SD of three repeated cell assays. *P < 0.05; two-way ANOVA.

To explore the role of BRD4 in S-AKI, we treated LPS-stimulated HK-2 cells with the BRD4 inhibitor JQ1. Based on prior studies, we used 10 μM JQ1, which is non-toxic and effective [23]. JQ1 treatment was associated with a reduction in LPS-induced BRD4 and NOX4 mRNA and protein levels (Fig. 5A, B). It also reversed the LPS-induced increase in NGAL, KIM-1, IL-1β, and TNF-α (Fig. 5C). Furthermore, MDA and ROS levels were significantly decreased by JQ1 (Fig. 5D–F). These results suggest that BRD4 inhibition suppresses inflammation and oxidative stress, at least partly through downregulating NOX4.

### BRD4 inhibition suppresses NOX4 expression and mitigates inflammation and oxidative stress in mice

**Fig. 6:**
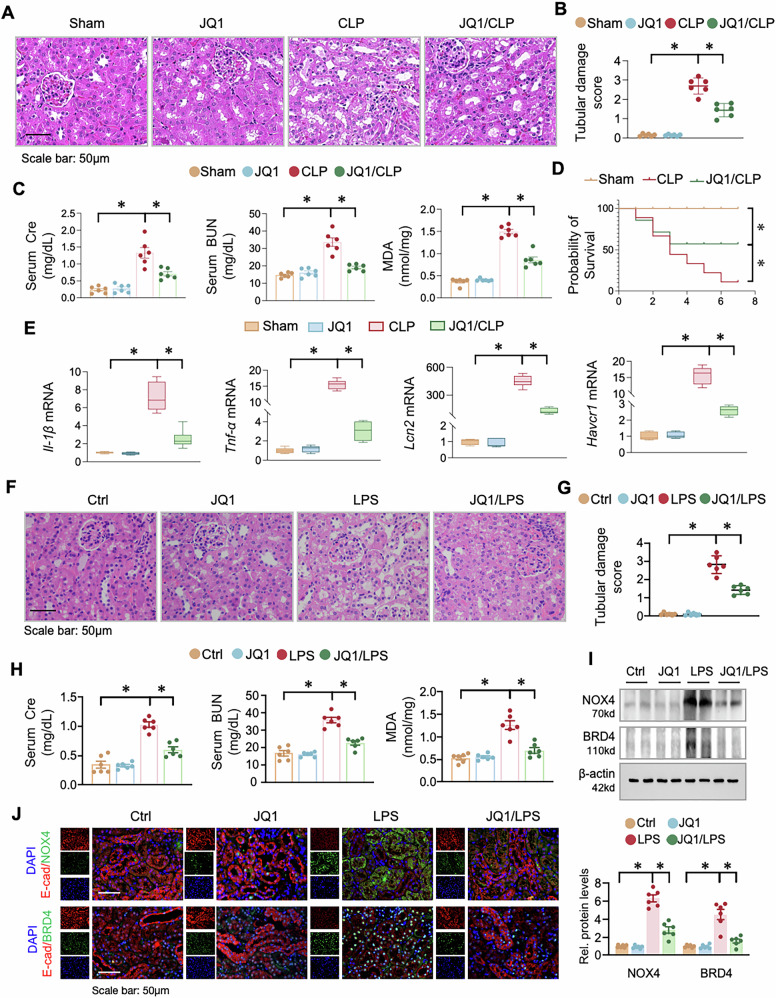
BRD4 inhibition reduce the expression of NOX4 and block inflammation and oxidative stress in mice. C57BL/6 mice were pretreated with JQ1 or vehicle 1 hour before the administration of CLP or Sham (*n* = 6). **A** Representative photomicrographs of kidney sections from above mice stained by Hematoxylin-eosin staining (H&E). **B** Quantification of tubular damage based on (**A**). **C** Serum creatinine (Cre), blood urine nitrogen (BUN) and renal malondialdehyde (MDA) levels were measured in the experimental mice indicated above. **D** The survival rate was compared among Sham, CLP, JQ1/CLP group by Kaplan–Meier test (*n* = 10 for each group). **E** QRT-PCR of *Il-1β, Tnf-α, Lcn2 and Havcr1* mRNAs in renal tissues from above mice. C57BL/6 mice were pretreated with JQ1 (50 mg/kg, i.p. once, *n* = 6) or vehicle 1 hour before the administration of LPS or control vehicle. **F** Representative photomicrographs of kidney sections from above mice stained by H&E. **G** Quantification of tubular damage based on (F). **H** Cre, BUN and renal MDA levels were measured in the experimental mice indicated above. **I** Western blots. The renal tissues were assayed for NOX4 and BRD4. Two samples from each group were shown. The quantification was presented in the lower panel. **J** Representative immunofluorescence images showing co-localization of NOX4 or BRD4 (green) with the epithelial marker E-cadherin (E-cad, red) in kidney sections from control and LPS-treated mice with or without JQ1 treatment. Nuclei were stained with DAPI (blue). Data were presented as means ± SEM, *P < 0.05, two-way ANOVA.

To further evaluate the effect of BRD4 inhibition in vivo, mice were treated with JQ1 in both CLP-induced and LPS-induced models. In the CLP model, JQ1 treatment alone did not affect normal kidney morphology but significantly attenuated CLP-induced tubular injury (Fig. 6A, B). Consistently, JQ1 ameliorated CLP-induced renal dysfunction and oxidative stress, indicated by significant reductions in serum Cre, BUN, and MDA levels (Fig. 6C). What’s more, JQ1 improved survival in the CLP model and reversed the mRNA induction of NGAL, KIM-1, IL-1β, and TNF-α (Fig. 6D, E).

In the LPS model, JQ1 pretreatment similarly attenuated LPS-induced renal histological damage (Fig. 6F, G), and improved renal function and oxidative stress (Fig. 6H). At the molecular level, JQ1 treatment was associated with reduced protein expression of BRD4 and NOX4, as determined by western blotting and IHC (Fig. 6I, Supplementary Fig. S1A and B), and decreased mRNA levels of BRD4, NOX4 and the same panel of injury and inflammatory markers (Supplementary figure S1C, D). Immunofluorescence double staining further confirmed that BRD4 and NOX4 induction was prominently localized to renal tubular epithelial cells (E-cad-positive) during S-AKI, and this epithelial-associated upregulation was effectively reversed by JQ1 treatment (Fig. 6J).

Taken together, these convergent results from two distinct experimental models of sepsis demonstrate that BRD4 inhibition exerts potent renoprotective effects, which are mediated, at least in part, through the downregulation of NOX4 and subsequent mitigation of inflammatory and oxidative stress pathways.

### BRD4 promotes NOX4 transcription via P300-dependent histone acetylation

**Fig. 7:**
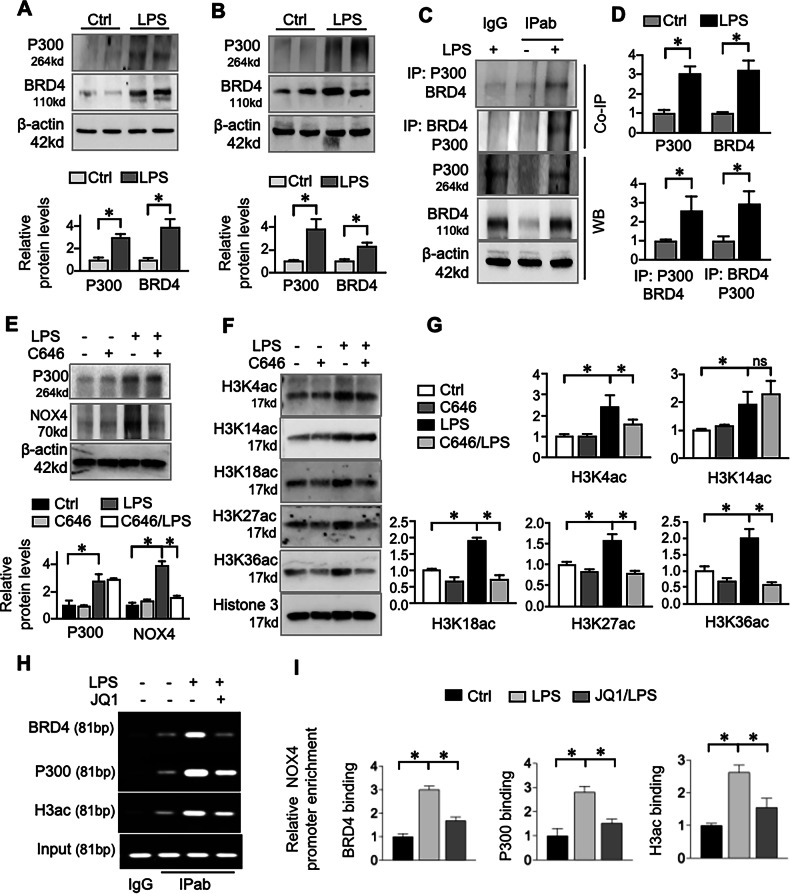
BRD4 promotes NOX4 via P300-mediated histone acetylation. **A** Western blots. HK2 cells were subjected to LPS (500 ng/mL) for 24 h and then were assayed for P300 and BRD4. The lower panel is the quantification. Data were presented as means ± SD of three repeated cell assays. *P < 0.05, one-way ANOVA. **B** Western blots. Renal tissues from control (Ctrl) and LPS group mice were assayed for P300 and BRD4. The lower panel is the quantification. Data were presented as means ± SEM, *n* = 6, *P < 0.05, one-way ANOVA. **C** Co-IP assay. Renal tissues of control (Ctrl) and LPS group mice were tested for P300 and BRD4. The same tissue lysates were immunoprecipitated with isoform-matched immunoglobulin (IgG) or antibody to P300 and BRD4, and then immunoprecipitants were assessed for P300 and BRD4 by western blotting. **D** Quantification of Co-IP. Data were presented as means ± SEM based on three renal samples from each group. *P < 0.05, one-way ANOVA. **E** Western blots. HK2 cells were treated with LPS (500 ng/mL) in presence or absence of C646 (10 μM) for 24 h, and then the cell lysates were analyzed for P300 and NOX4 expression. Quantification represented densitometric analysis normalized to the corresponding loading controls from three independent experiments. Data were presented as means ± SD. *P < 0.05, two-way ANOVA. **F** HK2 cells were treated with LPS (500 ng/mL) in presence or absence of C646 (10 μM) for 24 h, and then the cell lysates were tested for H3K4ac, H3K14ac, H3K18ac, H3K27ac and H3K36ac. Total Histone 3 served as controls. **G** Quantifications of protein levels in (F). Data were presented as means ± SD of three repeated cell assays. *P < 0.05, two-way ANOVA. **H, I** ChIP assay. HK2 cells were treated with LPS (500 ng/mL) with or without JQ1 (10 μM) for 4 h. ChIP assay was performed with antibodies to BRD4, P300, acetylated histone H3 (H3ac) or isoform-matched IgG, respectively. The immunoprecipitated DNA were analyzed by regular PCR and qRT-PCR. The PCR products were analyzed on an agarose gel (left panel). qRT-PCR (right panel) was normalized to input DNA and presented as fold changes relative to Ctrl. Data were presented as means ± SD of three repeated cell assays. Data were presented as means ± SD of three repeated cell assays. *P < 0.05, one-way ANOVA.

Having established that BRD4 inhibition downregulates NOX4 expression, we sought to elucidate the underlying transcriptional mechanism. BRD4 is known to activate transcription by recruiting transcriptional coactivators such as the histone acetyltransferase P300 [29, 30]. We hypothesized that P300 mediates the transcriptional activation of NOX4 by BRD4 in S-AKI. We first observed increased BRD4 and P300 levels in both LPS-treated HK2 cells (Fig. 7A) and mouse kidneys (Fig. 7B). Co-immunoprecipitation assays confirmed a physical interaction between BRD4 and P300 in the kidneys of LPS-induced mice (Fig. 7C, D). To assess the functional requirement of P300, we used the inhibitor C646. Treatment with C646 suppressed LPS-induced NOX4 expression (Fig. 7E). In addition, LPS stimulation broadly increased histone acetylation at H3K4, H3K14, H3K18, H3K27, and H4K36 sites. C646 treatment reversed acetylation at H3K4, H3K18, H3K27, and H4K36 (Fig. 7F, G), identifying these as P300-sensitive modifications in the S-AKI kidney. To obtain direct evidence that this complex acts at the NOX4 promoter, we performed ChIP assays. In LPS-stimulated HK2 cells, we found a significant enrichment of BRD4, P300, and acetylated histone H3 (H3ac) specifically at the NOX4 promoter region. Critically, this enrichment was markedly attenuated by pretreatment with the BRD4 inhibitor JQ1 (Fig. 7H, I). Collectively, these findings demonstrate that BRD4 facilitates P300 recruitment to the NOX4 promoter, enhances histone acetylation at specific loci, and thereby directly activates NOX4 transcription during S-AKI.

### Suppression of NOX4 is essential for the protective effects of BRD4 inhibition

**Fig. 8:**
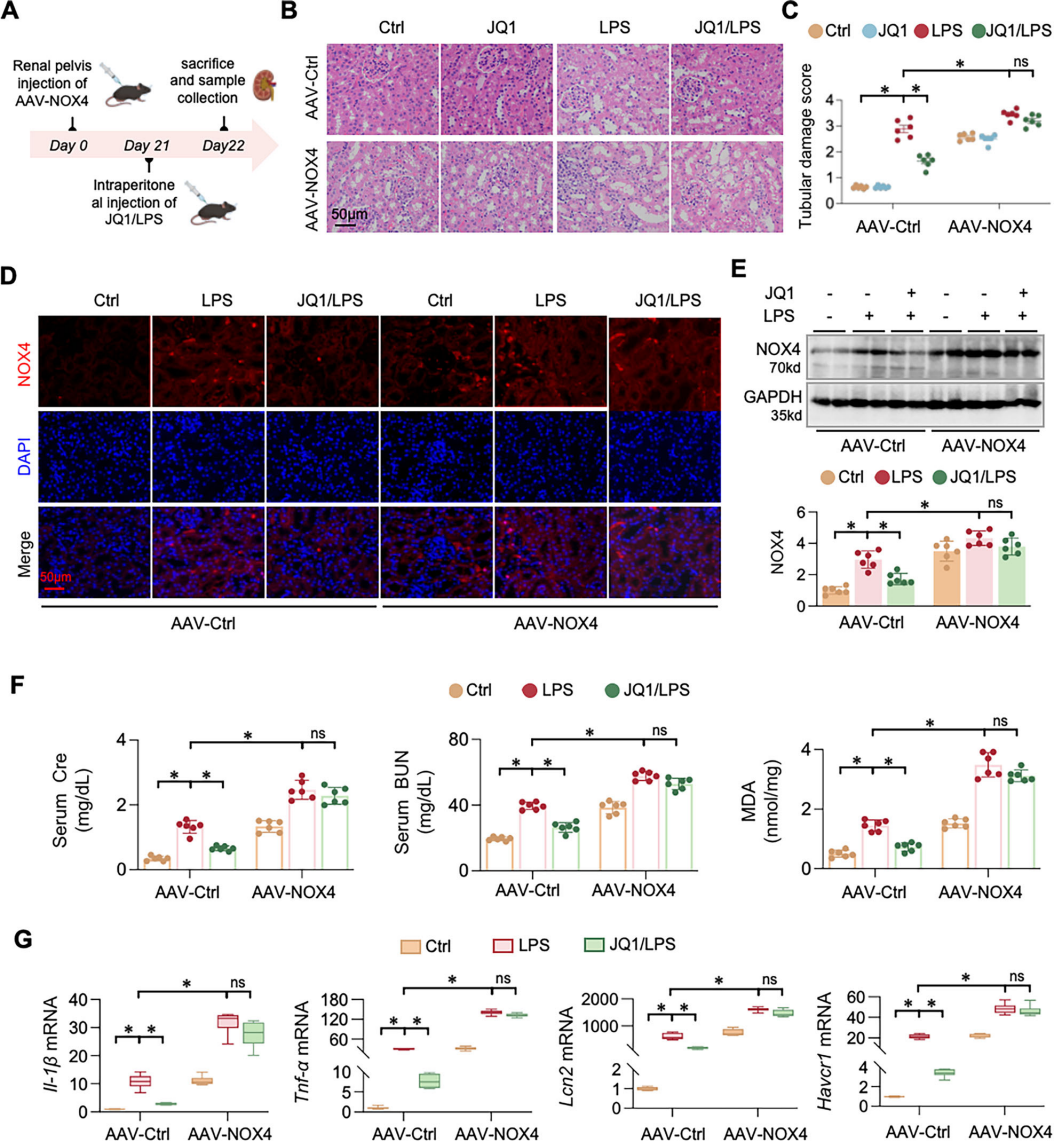
Suppression of NOX4 expression is essential for the protective effects of BRD4 inhibition in vivo. **A** Schematic diagram of the animal experiment design and process. Mice receiving AAV-Ctrl or AAV-NOX4 were subgrouped into control (Ctrl), JQ1, LPS, and JQ1-treated LPS (*n* = 6). **B** Representative photomicrographs of kidney sections stained by Hematoxylin-eosin staining (H&E). **C** Quantification of (B). *P < 0.05, three-way ANOVA followed by Tukey’s post-hoc test. **D** Representative photomicrographs of kidney sections from the experimental mice indicated above stained for NOX4 and DAPI by immunofluorescence (IF) staining. **E** The western blot of NOX4 protein in mouse kidney tissues. The lower panel was the quantification. **F** Serum creatinine (Cre), blood urine nitrogen (BUN) and renal malondialdehyde (MDA) levels were measured in the experimental mice indicated above. **G** QPCR of *Il-1β, Tnf-α, Lcn2* and *Havcr1* mRNAs in renal tissues. Data were presented as means ± SEM, *P < 0.05, *n* = 6, two-way ANOVA.

To confirm whether NOX4 suppression is necessary for BRD4 inhibition- mediated renal protection, we overexpressed NOX4 in mice by injecting AAV-NOX4 into the renal pelvis. Immunofluorescence staining for EGFP three weeks after injection confirmed robust and efficient transduction that was predominantly observed in renal tubular cells (Supplementary Fig. S2), verifying the efficiency of AAV9-mediated gene delivery. Following this validation, the mice were subjected to control (Ctrl), JQ1, LPS, or JQ1 plus LPS treatment (JQ1/LPS) groups (Fig. 8A). As expected, AAV-NOX4 mice exhibited significantly increased NOX4 expression (Fig. 8D, E) and aggravated renal damage. JQ1 reduced renal injury in AAV-Ctrl mice, but this protective effect was largely abolished in AAV-NOX4 mice (Fig. 8B, C). Consistently, JQ1 failed to significantly reduce serum Cre, BUN, and MDA levels in the AAV-NOX4 group (Fig. 8F). Similarly, JQ1-induced reductions in NGAL, KIM-1, IL-1β, and TNF-α mRNA levels were reversed by NOX4 overexpression (Fig. 8G). These data support that NOX4 downregulation is an important mediator of BRD4 inhibition–induced renal protection in S-AKI.

## Discussion

This study identifies BRD4 as a pivotal upstream epigenetic regulator that drives oxidative stress and inflammation during sepsis-associated acute kidney injury (S-AKI) by transcriptionally activating NOX4. We demonstrated that BRD4 and NOX4 are markedly upregulated in patients with S-AKI and in experimental models. Pharmacological inhibition of either protein significantly alleviated renal injury, oxidative stress, and inflammatory responses. Mechanistically, BRD4 enhances NOX4 transcription by recruiting the histone acetyltransferase P300, which mediates histone H3 acetylation at the NOX4 promoter. Importantly, the renoprotective effect of BRD4 inhibition was largely abolished by NOX4 overexpression, strongly support the BRD4-P300-NOX4 axis as a central driver of S-AKI pathogenesis. These findings uncover a previously unrecognized epigenetic mechanism linking BRD4 activation to NOX4-mediated oxidative injury and suggest that targeting BRD4 may represent a novel therapeutic strategy for S-AKI.

S-AKI is a common and severe complication of sepsis, characterized by multifactorial pathogenesis in which oxidative stress and inflammation play crucial roles. Oxidative stress leads to excessive production of reactive oxygen species (ROS), which damages renal tubular epithelial cells and promotes cell apoptosis and necrosis [9]. Concurrently, systemic inflammatory responses in sepsis trigger the release of large amounts of cytokines that exacerbate kidney injury [31]. Recent studies have highlighted signaling pathways that coordinate these responses, such as NF-κB, which amplifies inflammatory gene expression [32, 33], and TNF-α, which directly promotes renal inflammation and apoptosis [34, 35]. Conversely, activation of antioxidant transcription factors such as NFE2L2 (Nrf2) [36, 37] mitigates oxidative injury. Consistent with these concepts, we found that NOX4 levels are elevated in the serum of S-AKI patients and correlate positively with renal dysfunction. Experimental inhibition of NOX4 markedly attenuated oxidative stress, inflammatory cytokine expression, and renal injury, reinforcing its pathogenic role and therapeutic potential in S-AKI.

NOX4 expression is tightly regulated at multiple levels. Under hypoxic conditions, HIF-1α activates NOX4 transcription by binding to its promoter [38], while TGF-β and the PI3K/AKT pathway modulate NOX4 through Smad signaling and survival mechanisms [39, 40]. Recently, epigenetic modifications such as histone acetylation and DNA methylation have merged as key regulators of NOX4 gene expression [13, 41, 42]. Our study identified BRD4 as a novel epigenetic modulator of NOX4 in S-AKI. BRD4 expression was significantly upregulated in the LPS-induced S-AKI model and paralleled NOX4 induction. Pharmacological inhibition of BRD4 reversed NOX4 upregulation, attenuated inflammation and oxidative stress, and improved renal function, supporting the essential role of BRD4 in S-AKI pathogenesis. It should be noted that the observed reduction in BRD4 mRNA following JQ1 treatment likely reflects an indirect or context-dependent effect under inflammatory conditions, rather than a direct transcriptional inhibition of BRD4 by JQ1, which is primarily characterized as a bromodomain inhibitor.

It is noteworthy that pharmacological inhibition of NOX4 by GLX351322 not only suppressed its enzymatic activity but was also accompanied by a reduction in NOX4 expression in our S-AKI models. Although GLX351322 is primarily characterized as a selective inhibitor of NOX4-derived ROS production, similar decreases in NOX4 expression following GLX351322 treatment have been reported in other pathological contexts, including sepsis-induced lung injury and acute ocular hypertension [43, 44]. A plausible explanation for this phenomenon involves a ROS-dependent positive feedback mechanism regulating NOX4 transcription. NOX4-derived ROS can activate redox-sensitive transcription factors such as NF-κB, which has been shown to bind to the NOX4 promoter and further enhance its transcription, thereby forming a self-amplifying NOX4-ROS-NF-κB loop [45]. Pharmacological suppression of NOX4 activity by GLX351322 is therefore likely to disrupt this feedback circuit, leading to reduced ROS levels, attenuated NF-κB signaling, and secondary downregulation of NOX4 expression, thereby amplifying its protective effects against oxidative stress and inflammation in S-AKI.

Mechanistically, BRD4 acts as a transcriptional regulator that recognizes acetylated histones and recruits transcriptional co-activators such as p300/CBP [17, 46]. P300 catalyzes histone acetylation, thereby maintaining chromatin accessibility and stabilizing BRD4 occupancy at target loci. In our study, BRD4 promoted NOX4 transcription through P300-dependent histone H3 acetylation. Co-immunoprecipitation confirmed the interaction between BRD4 and P300, and inhibition of P300 by C646 reduced histone acetylation at specific lysine residues (H3K4, H3K18, H3K27, and H3K36) and reduced NOX4 expression. These results highlight P300 as an essential cofactor mediating BRD4-driven NOX4 activation in S-AKI. Although our findings define this BRD4-P300-NOX4 pathway, other transcriptional or epigenetic regulators may also contribute and warrant future investigation. A limitation of this study is that the AAV vector used to overexpress NOX4 was driven by a ubiquitous promoter, which does not allow definitive attribution of the observed effects exclusively to renal epithelial cells. Although immunofluorescence analysis indicated that NOX4 overexpression was predominantly localized to renal tubular epithelial cells, potential contributions from other renal cell types cannot be fully excluded. Future studies employing epithelial cell–specific promoters or conditional genetic approaches will be necessary to definitively establish cell type-restricted functions of NOX4.

In conclusion, our findings establish a mechanistic and translational link between BRD4-mediated epigenetic activation and NOX4-dependent oxidative injury in S-AKI. Pharmacological inhibition of BRD4 or NOX4 confers robust renoprotection by dampening inflammation and oxidative stress. Moreover, the positive correlation between circulating BRD4/NOX4 levels and renal dysfunction in patients underscores the clinical relevance of this pathway. Collectively, these data suggest that targeting BRD4-dependent transcriptional activation of NOX4 may provide a mechanistically guided therapeutic approach for the prevention and treatment of S-AKI.

## Materials and methods

### Human samples

Human serum samples (*n* = 27) were collected from patients who has sepsis complicated with acute kidney injury (*n* = 17) at the Intensive Care Unit or Department of Nephrology and the health checkup people (*n* = 10) in the Yancheng First People’s Hospital between January 1, 2023, and December 31, 2023. Basic demographic data, renal function parameters (serum creatinine, urea), and routine laboratory indices (PLT, WBC, NEUT, ALC, TBIL) were collected from the hospital information system at the time of sample collection. Sepsis severity was assessed by SOFA and GCS scores. Detailed baseline characteristics are summarized in Table 1. The study was approved by the Ethics Committee of Yancheng First People’s Hospital (No: 2023-K-161) and informed consent was obtained from all subjects.

### Enzyme-linked immunosorbent assay (ELISA)

The levels of BRD4 and NOX4 in the human serum were detected with human BRD4 ELISA kit (EH14093, FineTest, China) and human NOX4 ELISA kit (EH1662, FineTest, China) following the manufacturer’s instructions. The absorbance values of each sample were measured at 450 nm in a microplate reader (Varioskan™ LUX, Thermo Fisher Scientific, USA).

### Animal study

Eight-week-old male C57BL/6 mice (20–25 g) were procured from Junke Biological Engineering Co., Nanjing, China). Use of animal and the experimental procedures were in accordance with the Institutional Animal Care and Use Committee guidelines and approved by the Animal Care Committee of Jiangsu Vocational College of Medicine (No: SYXK2023-0005). Mice were housed under 25 ± 2 °C temperature, 50 ± 60% humidity and a 12-h light/dark cycle conditions with ad libitum access to food and water.

Two mouse models of S-AKI were established using 8-week-old male mice. The first model was induced by a single intraperitoneal injection of LPS (L2630, Sigma-Aldrich, USA) at a dose of 10 mg/kg, while control mice received an equal volume of vehicle. The second model was established using the cecal ligation and puncture (CLP) procedure [47]. Briefly, mice were anesthetized, followed by a midline laparotomy. The cecum was exposed, ligated, and punctured with a needle to induce polymicrobial sepsis. The abdominal incision was subsequently closed in layers. Sham-operated control animals underwent identical surgical procedures excluding ligation and puncture. All mice received appropriate postoperative fluid resuscitation and analgesia.

All mice were randomly divided into experimental groups as detailed below. Each group contained six mice. In the LPS-induced model, the groups were: (1) vehicle control (Ctrl); (2) JQ1 injection (HY13030, MedChemExpress, USA, 50 mg/kg intraperitoneal injection) or GLX351322 (HY-100111, MedChemExpress, USA, 5 mg/kg intraperitoneal injection); (3) LPS treatment; (4) JQ1 or GLX351322 intervention in LPS-treated mice (pretreatment for one hour followed by LPS administration) [44, 48]. In the CLP-induced model, the groups were: (1) sham-operated control (Sham); (2) JQ1 or GLX351322 alone (doses as above); (3) CLP surgery; (4) JQ1 or GLX351322 intervention of CLP mice (pretreatment for one hour followed by CLP). For survival analysis in the CLP model, mice were randomly assigned to three groups (*n* = 10 per group): Sham, CLP and JQ1/CLP. JQ1 (50 mg/kg, intraperitoneally) was administered one hour prior to CLP surgery and subsequently once daily for 7 consecutive days. Survival was monitored and recorded daily for 7 days following CLP.

For study to determine the role of NOX4 in the JQ1 intervention, C57BL/6 mice were injected through renal pelvis injection with either a control adeno-associated virus 9 (AAV) or NOX4 overexpression AAV (refer to NOX4 overexpression AAV and renal pelvis injection) three weeks before the experiments and then subjected to control, JQ1, LPS and JQ1/LPS. At least six animals were included in each group to ensure the effect size. The animals were sacrificed at 24 h after LPS injection. Blood samples were obtained at sacrifice via cardiac puncture, and the mouse kidneys were surgically collected, fixed for histological assay or stored at −80 °C for further analysis.

### NOX4 overexpression AAV and renal pelvis injection

The control and NOX4 overexpression adeno-associated virus serotype 9 (AAV-Ctrl and AAV-NOX4; #GOSV5011619) were generated by GeneChem Co. Ltd. (Shanghai, China) using the CMV-MCS-3Flag-FT2A-EGFP-WPRE-BGH polyA vector. A single dose of 50 μL AAV-Ctrl or AAV-NOX4 (1.5 ×1012 vg/mL) was injected into the renal pelvis of mice, and experiments were performed three weeks later when transgene expression reached its peak. For AAV administration, mice were anesthetized with tribromoethanol via intraperitoneal injection, and the kidneys were exposed through a small dorsal incision. A total of 50 μL of AAV solution was gently injected into the renal pelvis using an insulin syringe, followed by slow withdrawal of the needle and compression of the injection site with a sterile cotton swab for 3–5 min to prevent leakage. The kidney was then repositioned into the abdominal cavity, the incision was sutured, and the animals were maintained on a heating pad until full recovery [49]. Three weeks after AAV injection, LPS was intraperitoneally administered to induce S-AKI.

### Hematoxylin and Eosin staining (H&E)

Kidney tissues were fixed with 4% paraformaldehyde for paraffin embedding, stained with H&E and examined with an Olympus BX53 microscope. The tissue damage was scored in a blinded manner based on the proportion of damaged renal tubules and histological injury indicated by brush border loss, tubular dilation/flattening, tubular degeneration, tubular cast formation as well as vacuolization. Tissue injury was scored on a scale of 0–4, with 0 to 4 respectively representing 0%, <25%, 26–50%, 51–75% andå 76% of impaired renal tubules [50]. For each sample, ten randomly selected fields of ×400 amplification were examined and averaged.

### Immunohistochemistry staining

Immunohistochemistry (IHC) staining of kidney sections was performed essentially as before [51]. Briefly, tissue sections were dewaxed, dehydrated and then washed by PBS. After removing endogenous peroxidase with 3% H_2_O_2_, antigen retrieval was performed by applying citrate. The primary antibody anti-NOX4 (A11274, ABclonal, China) and anti-BRD4 (E1Y1P, Cell Signaling Technology, USA) was subjected to overnight incubation at 4 °C. Following PBS washings, biotinylated secondary antibody was used to incubate the slices at room temperature for 1 h and the sections were sealed with neutral resins and observed with an Olympus BX53 microscope (Tokyo, Japan). Mean density was quantified blindly using Image Pro Plus 6.0 software (Media Cybernetics, Inc., Rockville, MD, USA) to analyze the semiquantitative expression of NOX4 and BRD4.

### Immunofluorescence (IF) staining

For mice kidney tissues, paraffin-embedded mouse kidney sections (3 μm) were prepared by a routine procedure. The sections were deparaffinized, rehydrated and then heated in sodium citrate buffer. After blocking with goat serum, the slides were incubated with the following primary antibody overnight at 4 °C: anti-NOX4 (A11274, ABclonal, China), anti-BRD4 (E1Y1P, Cell Signaling Technology, USA) and anti-EGFP (GB1160, Servicebio, China). Next, the slides were incubated with fluorescently labeled secondary antibodies at room temperature for 1 h, and the nuclei were stained with DAPI. The sections were scanned with the Olympus SLIDEVIEW VS200 (Tokyo, Japan).

### Serum creatinine (Cre) and blood urea nitrogen (BUN) assay

The blood samples of experimental mice were centrifuged at 4000 rpm for 20 min at 4 °C to obtain serum. Serum creatinine (Cre) and blood urea nitrogen (BUN) levels (mg/dL) were performed using the Creatinine Assay kit (D799853, Sangon Biotech, China) and the Urea Nitrogen Assay kit (BC1535, Solarbio, China), respectively, according to the manufacturer’s assay protocols.

### Cell culture and treatment

Human kidney tubular HK2 cell line was purchased from the American Type Culture Collection (ATCC). The cell line was tested negative for mycoplasma contamination prior to use. Cells were cultured in DMEM/F12 medium (Corning, USA), supplemented with 10% fetal bovine serum and 1% penicillin/streptomycin (Gibco, USA) in a humidified 5% CO_2_ incubator at 37 °C, followed by stimulation with or without LPS (100, 500, 1000 ng/mL) for 24 h. JQ1 (10 μM) or C646 (10 μM, HY-13823, MedChemExpress, USA)was applied to pretreat HK2 cells at 1 h before LPS administration.

### Reactive Oxygen Species (ROS) measurement

Intracellular ROS levels were determined using 2’,7’-dichlorodihydrofluorescein diacetate (DCFH-DA, D6883, Sigma-Aldrich, USA) according to the instruction. Briefly, cells pre-treated with different reagents were incubated with 10 μM DCFH-DA for 30 min at 37°C and then observed by Olympus IX73 microscope (Tokyo, Japan). Mean fluorescence intensity was quantified blindly using Image J software (Media Cybernetics, Inc., Rockville, MD, USA) to analyze the ROS levels.

### Quantitative real-time PCR (qRT-PCR)

Total RNA was isolated from HK-2 cells or frozen kidney tissues using FreeZol Reagent (R711, Vazyme, China). Subsequently, it was purified and reverse-transcribed into cDNA using a HiScript II 1st Strand cDNA Synthesis Kit (R211, Vazyme, China). qRT-PCR analysis was performed using ChamQ Universal SYBR qPCR Master Mix (Q711, Vazyme, China). The relative mRNA levels were calculated using the 2^–ΔΔCT^ method with *Gapdh* serving as an internal reference. Primers for mRNAs of mouse genes and human genes were synthesized by Genscript Biotech Corporation (Nanjing, China) are listed in Table 2.

**Table 2:** Primer sequence (human (h) and mouse (m)).

Gene	Accession number	Primer sequence
h*IL-1β*	NM_000576.3	Forward: ATGATGGCTTATTACAGTGGCAA
		Reverse: GTCGGAGATTCGTAGCTGGA
h*TNF-α*	NM_000594.4	Forward: CCTCTCTCTAATCAGCCCTCTG
		Reverse: GAGGACCTGGGAGTAGATGAG
h*HAVCR1*	NM_001173393.3	Forward: TGGCAGATTCTGTAGCTGGTT
		Reverse: AGAGAACATGAGCCTCTATTCCA
h*LCN2*	NM_005564.5	Forward: GACAACCAATTCCAGGGGAAG
		Reverse: GCATACATCTTTTGCGGGTCT
h*BRD4*	NM_001330384.2	Forward: ACCTCCAACCCTAACAAGCC
		Reverse: TTTCCATAGTGTCTTGAGCACC
h*NOX4*	NM_001143836.3	Forward: CAGATGTTGGGGCTAGGATTG
		Reverse: GAGTGTTCGGCACATGGGTA
h*GAPDH*	NM_001256799.3	Forward: GTCCATGCCATCACTGCCAC
		Reverse: GCCTGCTTCACCACCTTCTTG
m*Il1-β*	NM_008361.4	Forward: GCTTCAGGCAGGCAGTATC
		Reverse: AGGATGGGCTC TTCTTCAAAG
m*Tnf-α*	NM_001278601.1	Forward: GACAAGCCTGTAGCCCACGT
		Reverse: ACAAGGTACAACCCATCGGC
m*Havcr1*	NM_001166631.1	Forward: TGTCGAGTGGAGATTCCTGGATGGT
		Reverse: GGTCTTCCTGTAGCTGTGGGCC
m*Lcn2*	NM_008491.1	Forward: GCAGGTGGTACGTTGTGGG
		Reverse: CTCTTGTAGCTCATAGATGGTGC
m*Brd4*	NM_001286630.1	Forward: ACCTCCAACCCTAACAAGCC
		Reverse: TTTCCATAGTGTCTTGAGCACC
m*Nox4*	NM_001285833.1	Forward: ACTGATCGCTCCTTTCGGTC
		Reverse: CCTAGGCCCAACATTTGGTGA
m*Gapdh*	NM_001289726.2	Forward: AACGACCCCTTCATTGAC
		Reverse: TCCACGACATACTCAGCA

### Western blotting

Western blotting assays of renal tissue or cell lysates were performed as described before [52]. In short, HK2 cells and renal tissues were lysed with RIPA buffer (P0013B, Beyotime, China) supplemented with phosphatase inhibitor (ST506, Beyotime, China) and protease inhibitor mixture (P1005, Beyotime, China) to obtain the total proteins. Protein concentration was measured with BCA Assay Kit (23225, Thermo Fisher Scientific, USA) and the lysates were analyzed by SDS-PAGE. After being transferred onto PVDF membranes (IPVH00010, Millipore, USA), the protein contents were probed using the following antibodies: anti-BRD4 (E1Y1P, Cell Signaling Technology, USA), anti-NOX4 (A11274, ABclonal, China), anti-P300 (D8Z4E, Cell Signaling Technology, USA); anti-H3K4ac (PTM-188), anti-H3K14ac (PTM-157), anti-H3K18ac (PTM-158), anti-H3K27ac (PTM-160), anti-H3K36ac (PTM-117), all from PTM BIO, China; anti-Histone H3 (A2348, ABclonal, China), anti-GAPDH (1E6D9, Proteintech, China) and anti-β-actin (AC026, ABclonal, China) served as controls. Goat anti-rabbit IgG-HRP (E-AB-1003, Elabscience, China) and goat anti-mouse IgG-HRP (E-AB-1001, Elabscience, China) were used as the secondary antibodies. Immunoblotting was visualized with ECL (ABclonal, Wuhan, China) using the ChemiDoc imaging system (Bio-Rad,California, USA), and protein bands were quantified with Image J software.

### Malondialdehyde (MDA) assay

Cell and renal tissue malondialdehyde (MDA) were measured with a Lipid Peroxidation MDA Assay Kit (KTB1050, Abbkine, China) according to the manufacturer’s instruction. The cell lysates and tissue homogenates were mixed with MDA reaction mix and boiled at 95 °C for 30 min, and then the absorbances at 532 nm and 600 nm were measured with a multifunctional microplate reader (Varioskan™ LUX, Thermo Fisher Scientific, USA). Data were expressed as the fold changes related to the control.

### Co-immunoprecipitation (Co-IP)

Kidney tissues were lysed in IP buffer (P0013, Beyotime, China). The kidney tissues lysates were first immunoprecipitated with antibody to BRD4 (E1Y1P, Cell Signaling Technology, USA) or P300 (D8Z4E, Cell Signaling Technology, USA), isoform-matched immunoglobulin (IgG, AC005, ABclonal, China) overnight and then incubated with Protein A + G Agarose (P2055, Beyotime, China) and washed. The immunoprecipitants were eluted and subjected to western blot analysis with antibodies to P300 or BRD4 respectively. The non-immunoprecipitated lysates served as the internal controls.

### Chromatin immunoprecipitation (ChIP)

ChIP assays were carried out using a ChIP Assay Kit (P2078, Beyotime, Shanghai, China). Three antibodies anti-BRD4 (E1Y1P, Cell Signaling Technology, USA), anti-P300 (D8Z4E, Cell Signaling Technology, USA), anti-acetylated histone 3 (39139, Active Motif, California, USA) or isoform-matched IgG (AC005, ABclonal, China) were used in the immunoprecipitation (IP) step. The IPed-DNAs and the input DNA (2% of IP) were analyzed by regular PCR and quantitative real time PCR (qRT-PCR). The primer sequences are Nox4p forward 5’-GGACATCCTGAACAGCAGCA-3’ (-230/-211) and Nox4p reverse 5’-CTGCACCAGTCTGCTCCG-3’ (-167/-150) [53]. The results were further analyzed on a 1% agarose gel for regular PCR or presented as the fold change relative to Ctrl for qRT-PCR, respectively.

### Statistical analysis

Data analyses were performed with GraphPad Prism 9.0 or SPSS 22.0. For human data, continuous variables are expressed as mean ± SD or median (interquartile range), as appropriate. Comparisons between groups were performed using the student’s t-test or Mann–Whitney U test for continuous data and the χ² test for categorical variables. For experimental data, one-way ADNOVA followed by Tukey’s post-hoc test was used to assess differences between two groups. For two or three factor experiments, data analyses were performed by two-way analysis of variance (ANOVA) or three-way ANOVA followed by Tukey’s post-hoc test. Data normality and the assumption of homogeneity of variances were determined by Shapiro–Wilk test and Levene’s test, respectively. Pearson’s test was used to analyze the correlation between normal distribution data. Spearman’s test was used to analyze the correlation between non-normal distribution data. Number of biological replicates is indicated in the figure legends. Statistically significance was defined as *P* < 0.05.

## Supplementary information

supplementary figure

uncropped western blots

## Data Availability

The RNA-sequencing data used in this study were obtained from the Gene Expression Repository (accession number: GSE227623). The original contributions presented in the study are included in the article, further inquiries can be directed to the corresponding author/s.
