# Supplementary material for: BRD4 Inhibition alleviates sepsis-associated acute kidney injury via suppression of NOX4-mediated oxidative stress and inflammation
Source: Cell Death Discov. 2026 Apr 21;12:266. doi: 10.1038/s41420-026-03113-y (PMC13230539; doi:10.1038/s41420-026-03113-y)
Supplement: Supplementary file 1 — supplementary figure [file 41420_2026_3113_MOESM1_ESM.pdf]

Supplementary figure S1

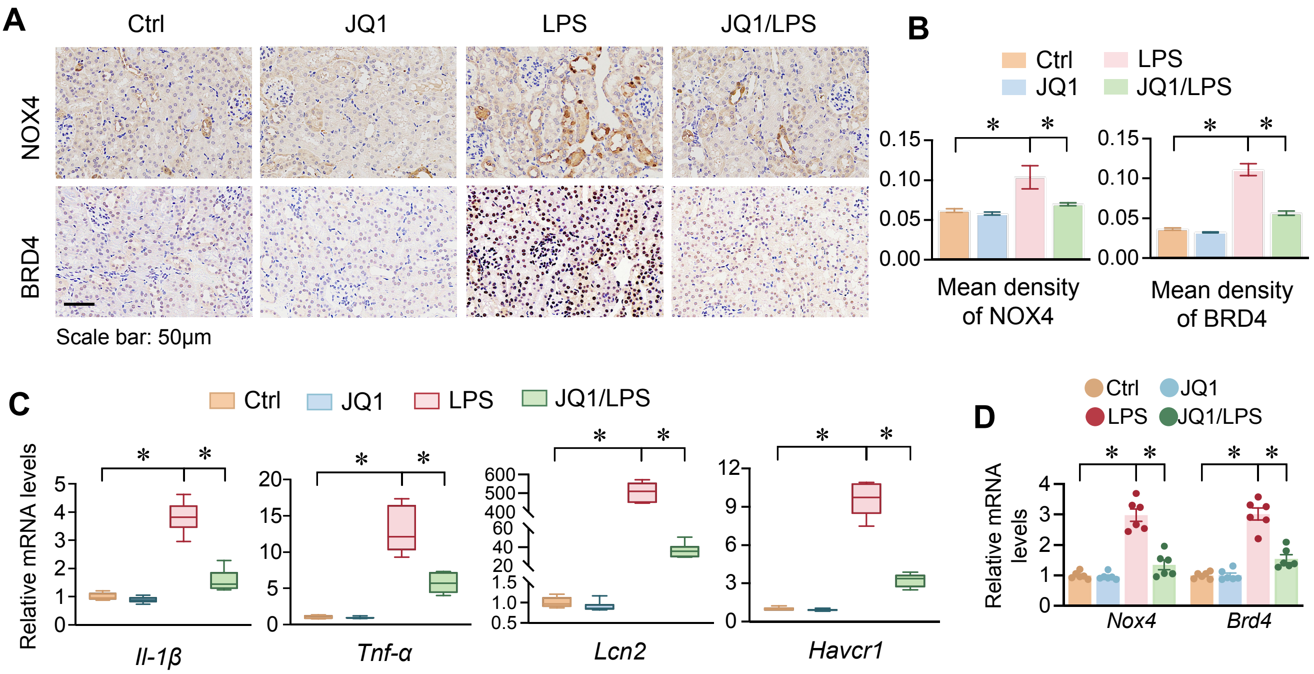

Supplementary figure S1. JQ1 attenuates inflammation in the LPS-induced model of S-AKI. C57BL/6 mice were pretreated with JQ1 or vehicle 1 hour prior to LPS or control vehicle administration. (A) Representative immunohistochemical staining of NOX4 and BRD4 in kidney sections from the indicated groups. (B) Quantification of f NOX4- and BRD4-positive staining areas. (C) QRT-PCR of *Il-1β, Tnf-α, Lcn2* and *Havcr1* mRNAs in renal tissues from the indicated groups. (D) QRT-PCR of *Nox4* and *Brd4* mRNAs in renal tissues from above group mice. Data were presented as means ± SEM, *$P < 0.05$, n = 6, two-way ANOVA.

Supplementary figure S2

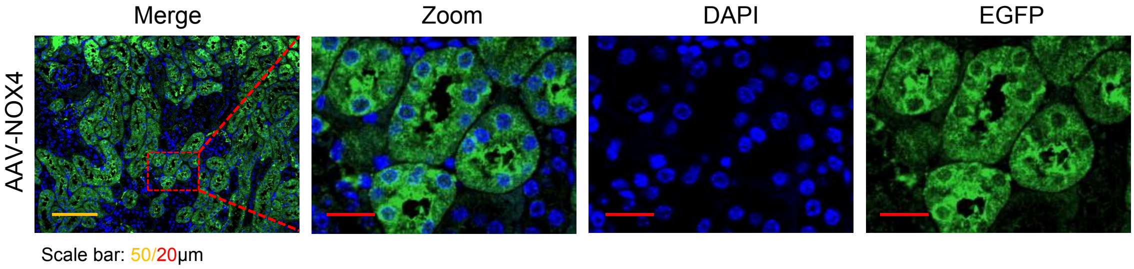

Supplementary figure S2. Validation of AAV9-mediated NOX4 overexpression in renal tubules. Mice were injected with AAV9 carrying a NOX4 overexpression construct and an EGFP reporter via the renal pelvis. Shown is a representative immunofluorescence image of kidney tissue collected three weeks post-injection, demonstrating robust EGFP signal (green) localized to renal tubules, confirming successful transduction.
