# Supplementary material for: BRD4 Inhibition alleviates sepsis-associated acute kidney injury via suppression of NOX4-mediated oxidative stress and inflammation
Source: Cell Death Discov. 2026 Apr 21;12:266. doi: 10.1038/s41420-026-03113-y (PMC13230539; doi:10.1038/s41420-026-03113-y)
Supplement: Supplementary file 2 — uncropped western blots [file 41420_2026_3113_MOESM2_ESM.pptx]

## Slide 1
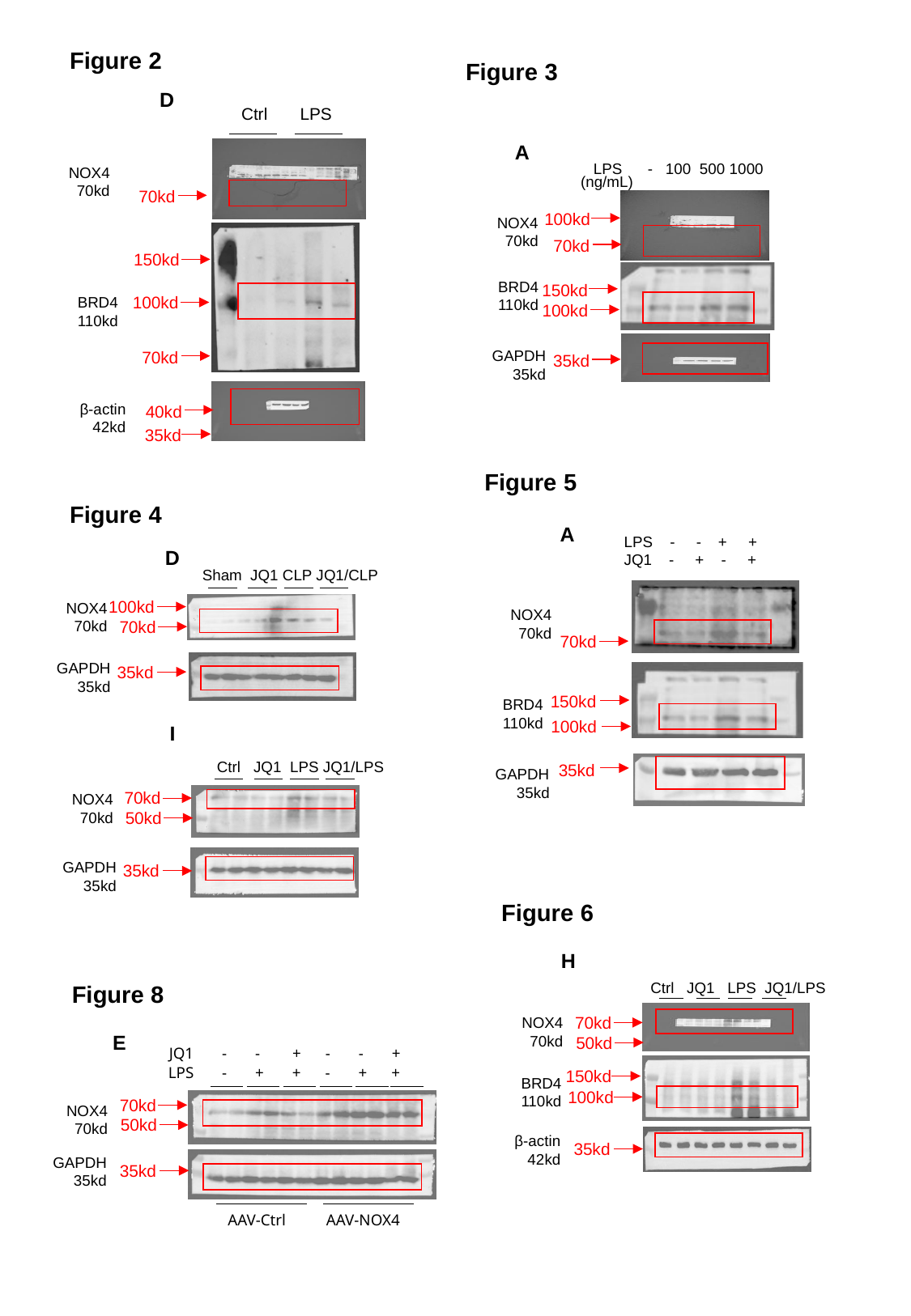

Figure 2
Figure 3
D
Ctrl LPS
A
NOX4
70kd
 LPS - 100 500 1000
(ng/mL)
70kd
100kd
NOX4
70kd
70kd
150kd
BRD4
110kd
150kd
100kd
BRD4
110kd
100kd
GAPDH
35kd
70kd
35kd
β-actin
42kd
40kd
35kd
Figure 5
Figure 4
A
LPS - - + +
JQ1 - + - +
D
Sham JQ1 CLP JQ1/CLP
100kd
NOX4
70kd
NOX4
70kd
70kd
70kd
GAPDH
35kd
35kd
150kd
BRD4
110kd
100kd
I
Ctrl JQ1 LPS JQ1/LPS
35kd
GAPDH
35kd
70kd
NOX4
70kd
50kd
GAPDH
35kd
35kd
Figure 6
H
Ctrl JQ1 LPS JQ1/LPS
Figure 8
70kd
NOX4
70kd
E
50kd
JQ1 - - + - - +
 LPS - + + - + +
150kd
BRD4
110kd
100kd
70kd
NOX4
70kd
50kd
β-actin
42kd
35kd
GAPDH
35kd
35kd
AAV-Ctrl AAV-NOX4

## Slide 2
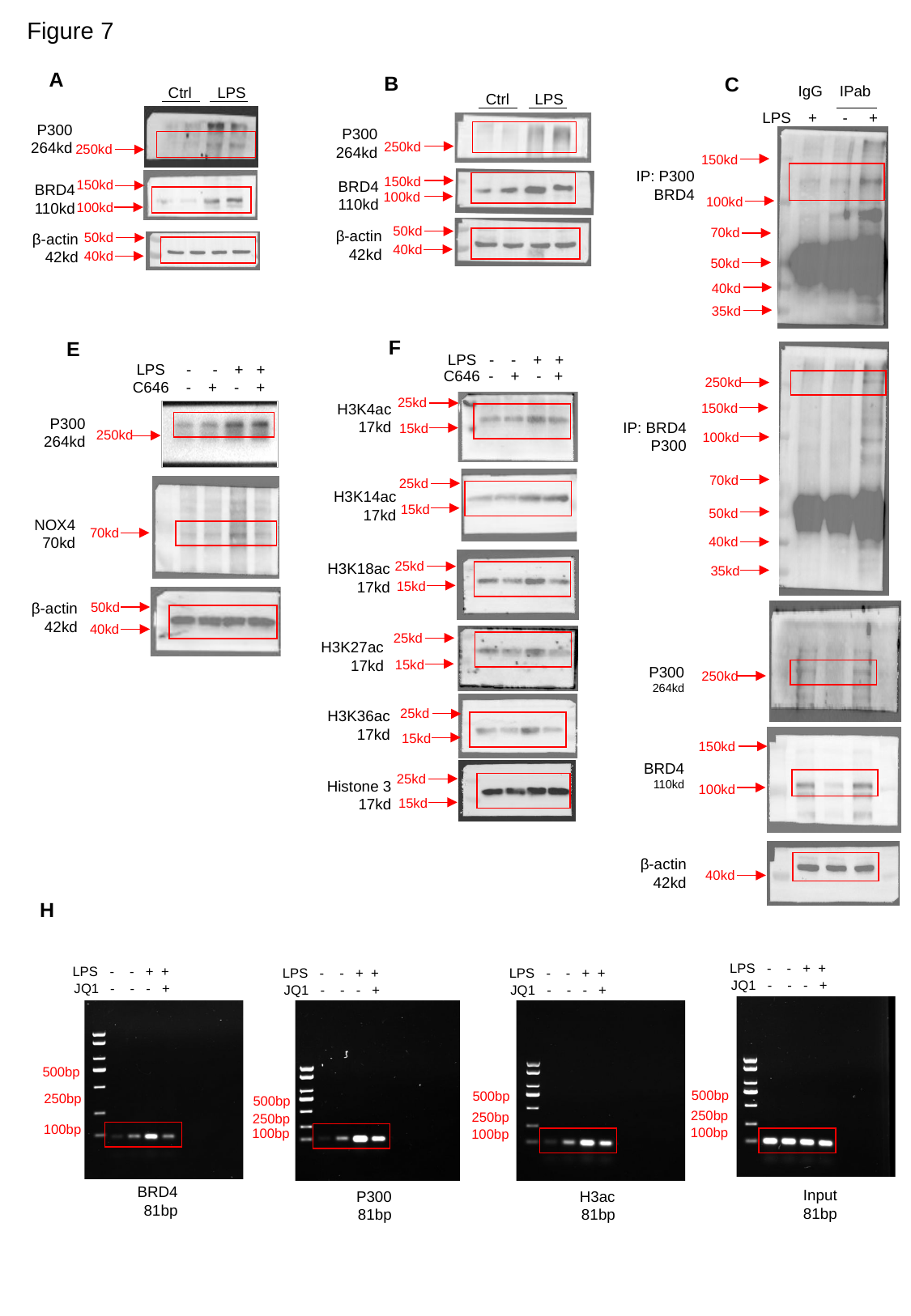

Figure 7
A
B
C
IgG IPab
Ctrl LPS
Ctrl LPS
LPS + - +
P300
264kd
P300
264kd
250kd
250kd
150kd
IP: P300
BRD4
150kd
150kd
BRD4
110kd
BRD4
110kd
100kd
100kd
100kd
50kd
70kd
β-actin
42kd
50kd
β-actin
42kd
40kd
40kd
50kd
40kd
35kd
F
E
LPS - - + +
LPS - - + +
C646 - + - +
250kd
C646 - + - +
25kd
150kd
H3K4ac17kd
P300
264kd
IP: BRD4
P300
15kd
250kd
100kd
70kd
25kd
H3K14ac
17kd
15kd
50kd
NOX4
70kd
70kd
40kd
25kd
H3K18ac
17kd
35kd
15kd
β-actin
42kd
50kd
40kd
25kd
H3K27ac
17kd
15kd
P300
264kd
250kd
25kd
H3K36ac
17kd
15kd
150kd
BRD4
110kd
25kd
Histone 3
17kd
100kd
15kd
β-actin
42kd
40kd
H
LPS - - + +
LPS - - + +
LPS - - + +
LPS - - + +
JQ1 - - - +
JQ1 - - - +
JQ1 - - - +
JQ1 - - - +
500bp
500bp
500bp
250bp
500bp
250bp
250bp
250bp
100bp
100bp
100bp
100bp
BRD4
81bp
Input
81bp
P300
81bp
H3ac
81bp
